# Assessing the Spatial Variability of Alfalfa Yield Using Satellite Imagery and Ground-Based Data

**DOI:** 10.1371/journal.pone.0157166

**Published:** 2016-06-09

**Authors:** Ahmed G. Kayad, Khalid A. Al-Gaadi, ElKamil Tola, Rangaswamy Madugundu, Ahmed M. Zeyada, Chariton Kalaitzidis

**Affiliations:** 1 Department of Agricultural Engineering, College of Food and Agriculture Sciences, King Saud University, Riyadh, Saudi Arabia; 2 Precision Agriculture Research Chair, King Saud University, Riyadh, Saudi Arabia; 3 Agricultural Engineering Research Institute (AEnRI), Agricultural Research Centre, Giza, Egypt; 4 Department of Geoinformation in Environmental Management, Mediterranean Agronomic Institute of Chania (MAICh), Chania, Greece; University of Vigo, SPAIN

## Abstract

Understanding the temporal and spatial variability in a crop yield is viewed as one of the key steps in the implementation of precision agriculture practices. Therefore, a study on a center pivot irrigated 23.5 ha field in Saudi Arabia was conducted to assess the variability in alfalfa yield using Landsat-8 imagery and a hay yield monitor data. In addition, the study was designed to also explore the potential of predicting the alfalfa yield using vegetation indices. A calibrated yield monitor mounted on a large rectangular hay baler was used to measure the actual alfalfa yield for four alfalfa harvests performed in the period from October 2013 to May 2014. A total of 18 Landsat-8 images, representing different crop growth stages, were used to derive different vegetation indices (VIs). Data from the yield monitor was used to generate yield maps, which illustrated a definite spatial variation in alfalfa yield across the experimental field for the four studied harvests as indicated by the high spatial correlation values (0.75 to 0.97) and the low P-values (4.7E-103 to 8.9E-27). The yield monitor-measured alfalfa actual yield was compared to the predicted yield form the Vis. Results of the study showed that there was a correlation between actual and predicted yield. The highest correlations were observed between actual yield and the predicted using NIR reflectance, SAVI and NDVI with maximum correlation coefficients of 0.69, 0.68 and 0.63, respectively.

## 1. Introduction

Alfalfa (*Medicago sativa*) is considered, worldwide, as being one of the most important forage crops. It is cultivated in more than 80 countries covering an area of 35 million hectares. Its growth structure is upright with crowns that contain 5–25 stems that grow up to 60 to 90 cm in height [1].

The amount or mass of the harvested crop per unit area, defined as being the crop yield [2], varies temporally or spatially within the same field [3]. Hence, knowledge of the spatial and the temporal variability in a crop yield is essential to help farm managers make the right decision in different situations [4], especially in precision agriculture (PA), as it provides detailed information about the final product of farmer’s practices [5,6]. In general, within-field variation in crop yield depends on the nature of the soil, the field topography, the amount of the applied agricultural inputs (fertilizers, irrigation water, etc.) and the patterns of irrigation and fertilizer practices [5]. Crop yield mapping is one of the essential precision agriculture tools that can provide a thorough understanding of yield variations across agricultural fields. Traditional biomass assessment methods, such as the quadratic frame sampling, are time-consuming, tedious and laborious when recording yield data [7]. These methods can be difficult, or even impossible, to practice in large areas. To overcome this difficulty, hay yield monitors have been developed and are widely used to measure hay yield during the baling process [8].

The advances in satellite-based remote sensing systems have provided a non-destructive, and an efficient means to predict yield in large, or even mega, commercial agricultural farms. Precision agriculture practices mainly rely on the combination of satellite-derived outputs, such as soil parameter and yield maps, and geostatistical techniques for the quantitative evaluation of within-field spatial variability [9]. In geospatial techniques, kriging is one of the most commonly used methods to generate estimated surfaces for the parameters under study, such as yield, soil nutrients, and nitrogen application rates, that are used in the characterization of agricultural fields [10, 11].

Several studies have used satellite remote sensing (RS) images and ground-based sensors as non-destructive tools for yield prediction in agricultural fields [7, 12, 13]. One of the two strategies that are widely used in crop yield prediction through RS data involves the use of this data in conjunction with plant physiological and meteorological models to predict crop development; hence, crop yield [13]. The second strategy, however, relies on the direct use of RS data to predict crop yield. The latter strategy works only if there is a direct relation between the spectral reflectance and the biomass. This is based on the fact that the reflectance decreases, in the red spectral region, and increases, in the near-infrared (NIR) region, as the vegetation density increases [14]. Multi-temporal images have been reported by a number of studies to be used for crop variability assessments using visible and NIR wavebands and vegetation indices (VIs). For instance, NIR waveband was used for the prediction of the biomass of forest stands [15], Mediterranean scrublands [16], grasslands [17], rice fields [18], cotton [14] and Alfalfa [19].

Spectral vegetation indices (VIs), calculated from reflectance detected by satellite images, which correlate the vegetation biophysical with spectral characteristics [20, 21, 22], are widely used for the prediction of crop yield and its spatial patterns across agricultural fields [7, 13]. Among the different VIs, the Normalized Difference Vegetation Index (NDVI), the Soil Adjusted Vegetation Index (SAVI) and the Green Normalized Difference Vegetation Index (GNDVI) have become popular indicators for studying vegetation health and crop production [23, 24]. The VIs that are related to different crop variables, such as plant cover, leaf area, chlorophyll and nitrogen content are commonly used to characterize crop growth performance [20, 25]. For instance, Trotter et al. [7] correlated the biomass of irrigated alfalfa to VIs, such as NDVI, SAVI and Simple Ratio (SR), by using an active sensor (crop circle). Various multispectral RS sensors have been used to estimate alfalfa hay yield, including SPOT and Landsat [26], AVHRR [13], Landsat and Quickbird [27] and AVIRIS hyperspectral data [28].

Most of the previous studies [26, 27] focused on only yield data on a large scale; however, small or field scale studies have been limited, particularly for alfalfa crop. Moreover, previous efforts for predicting alfalfa hay yield using VIs, with limited data at the field scale, resulted in poor response for both satellite images [13] and active optical sensors [7]. Although similar studies have been conducted across the world on alfalfa yield estimation using RS technology, such studies performed under the agricultural environment of Saudi Arabian region are very limited, introducing a lack of knowledge in the use of RS technology for studying different yield parameters. Therefore, this study conducted on a center pivot irrigated field in a dry environment was designed to: (i) generate alfalfa hay yield maps and assess the spatial variability in field productivity using a hay yield monitor data, (ii) assess the alfalfa yield, at different growth stages, through various vegetation indices (VIs) derived from landsat-8 satellite data and (iii) study the spatial correlation between actual alfalfa yield and the VIs and explore the possibility of developing an RS-based yield prediction system.

## 2. Materials and Methods

### 2.1 Study area

This study was conducted on a 23.5 ha field cultivated with alfalfa crop (Variety: *Green Master*), grown under a center pivot irrigation system in a commercial farm (Todhia Arable Farm—TAF) located between Al-Kharj and Haradh cities in the Eastern Province of Saudi Arabia (permission to conduct the study was issued by the Farm Manager Mr. Alan King). The TAF, which covers an area of 6,967 ha, lies within an arid climatic zone between the latitudes of 24°10' 22.77" and 24°12' 37.25" N and longitudes of 47°56' 14.60" and 48°05' 08.56" E. The soil in TAF is mainly sandy loam. Alfalfa crop was sown on November 26, 2012, and the study was initiated at a crop age of 11 months for the period from October 2013 to May 2014. Four consecutive cuts/harvests (numbered as 8, 9, 10 and 11) were studied.

### 2.2 Satellite Image Processing

**Fig 1:**
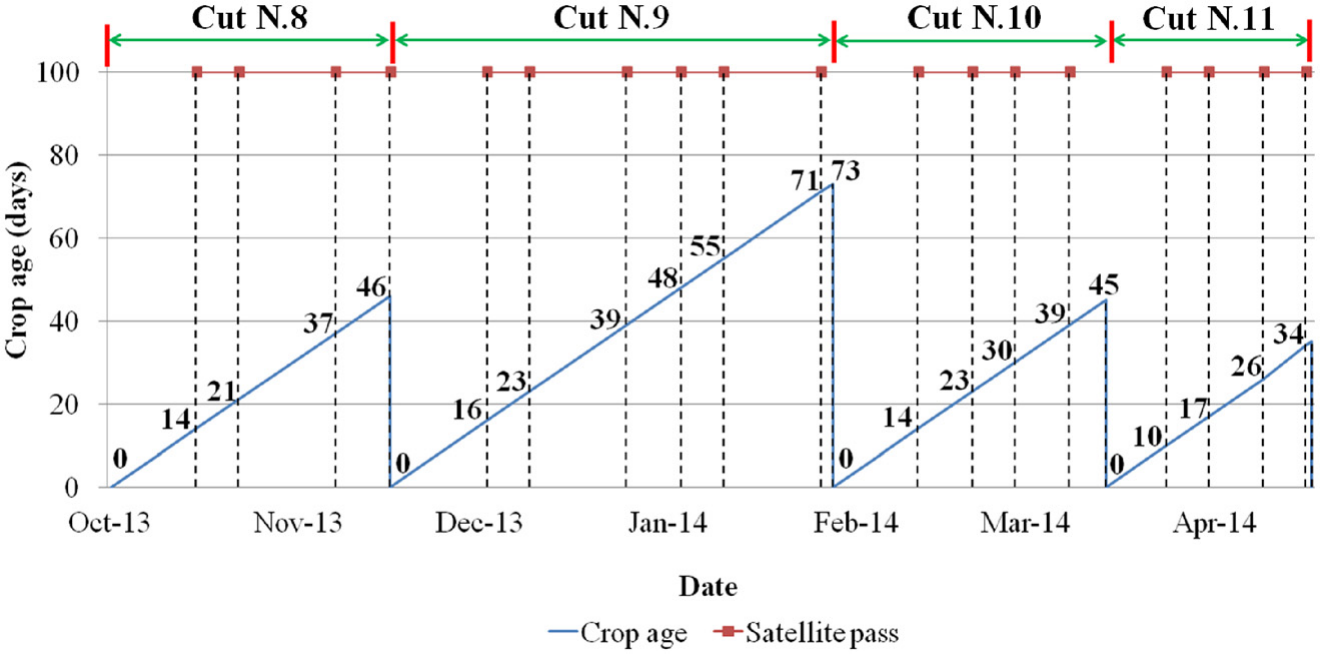
Dates of satellite images at different crop ages.

Cloud free Landsat-8 satellite images (Level 1 GeoTIFF data products) were downloaded from the Earth Explorer service (http://lpdaac.usgs.gov) of Land Processes Distributed Active Archive Center (LPDAAC), USGS/EROS, Sioux Falls, SD, USA. Dates of satellite images and the corresponding crop ages are shown in Fig 1.

**Fig 2:**
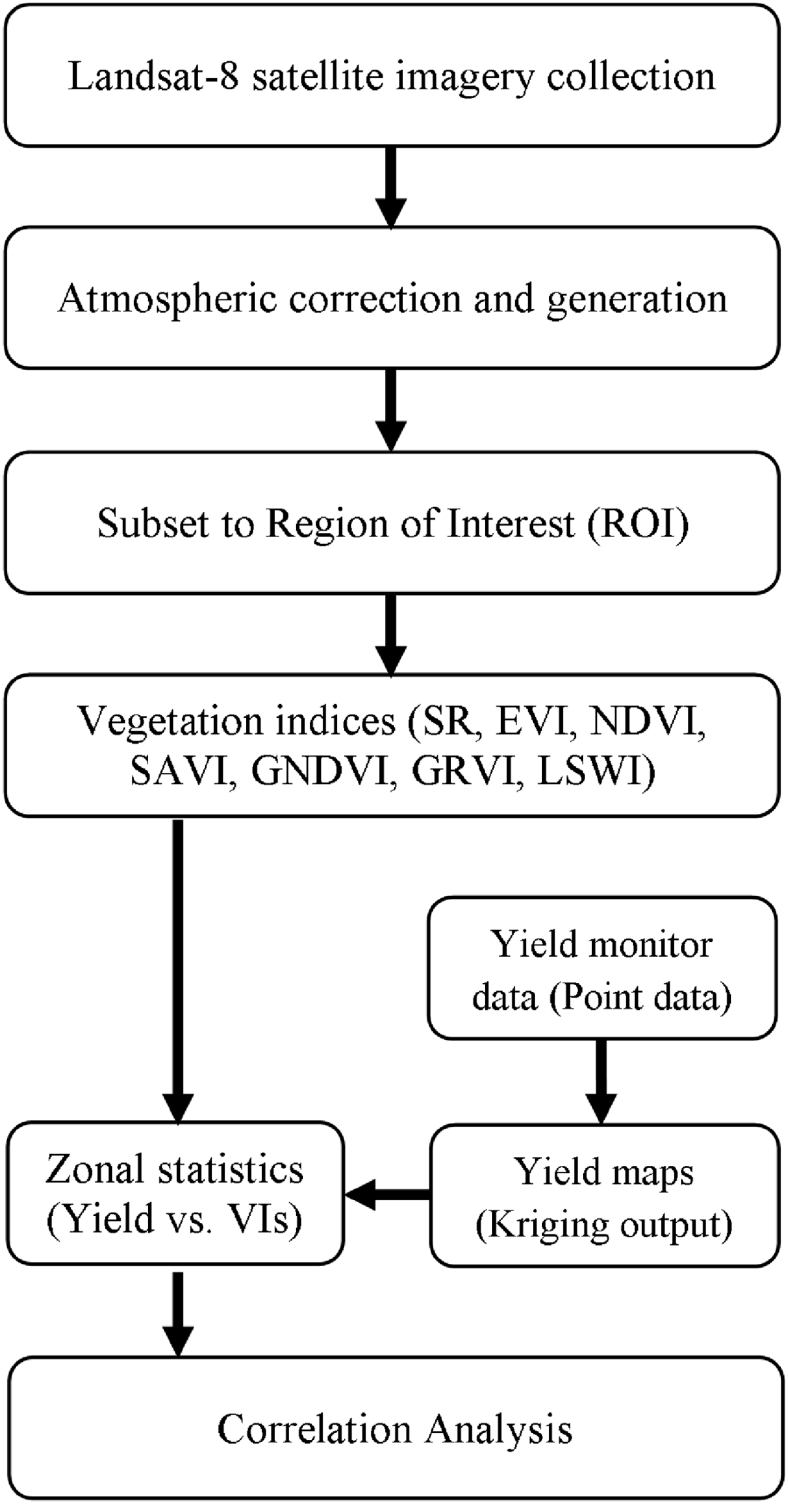
Image processing flow chart.

The level-1 Landsat-8 products were pre-processed to values of surface reflectance using Fast Line-of-sight Atmospheric Analysis of Spectral Hypercubes (FLAASH^®^), a radiometric calibration tool of ENVI software (Ver. 5.1, Research System Inc., Boulder, CO, USA). Region of Interest (ROI), the part of the center pivot field under the study, was extracted from the obtained reflectance images for further analysis. The steps followed in image analysis process are displayed in Fig 2.

### 2.3 Calculation of vegetation indices

The spectral indices, such as SR [29], Enhanced Vegetation Index (EVI) [30], NDVI [31], SAVI [32], Green Red Vegetation Index (GRVI) [33], GNDVI [34] and Land Surface Water Index (LSWI) [35] were calculated utilizing Eqs 1–7.

SR=(NIR/R) (1)

$$EVI=2.5\left[\frac{\left(NIR-R\right)}{\left(NIR+6R-7.5\;B+1\right)}\right]$$ (2)

NDVI=(NIR−R)/(NIR+R) (3)

$$SAVI=\frac{\left(NIR-R\right)}{\left(NIR+R+L\right)}\left(1+L\right)$$ (4)

GRVI=(G−R)/(G+R) (5)

GNDVI=(NIR−G)/(NIR+G) (6)

LSWI=(NIR−SWIR1)/(NIR+SWIR1) (7)

Where: R is Red, G is Green, B is Blue, NIR is Near Infrared and SWIR is Short Wave Infrared bands.

### 2.4 Cumulative vegetation indices

The acquired Landsat-8 images were analyzed for the reflectance at NIR channel and the aforementioned vegetation indices at different growth stages of alfalfa crop for the four studied harvests. The vegetation indices derived from the acquired Landsat-8 images were used to calculate the cumulative vegetation indices (CVIs) for each of the four harvests. To investigate the correlation between the obtained CVIs values and the corresponding actual alfalfa yield values, the collected observations were subjected to geospatial analysis.

### 2.5 Alfalfa yield monitoring

**Fig 3:**
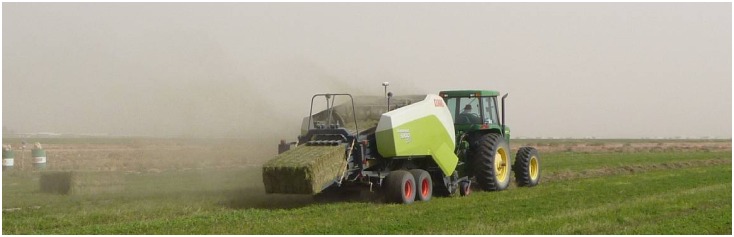
The hay baler, equipped with a hay yield monitor, at a baling process.

A large rectangular baler (CLAAS model Quadrant 3200), equipped with a hay yield monitoring system (model 500 of Harvest Tec), was used for recording, on-the-go, the alfalfa yield data at the time of baling (Fig 3). The monitoring system provided data on the hay mass flow rate, moisture content (MC) of the crop at baling, along with wet and dry hay yield (t ha^-1^) at a given geo-referenced location (GPS coordinates).

The MC, GPS and mass flow sensors were calibrated, and the results showed that the correlation between the actual alfalfa yield and that estimated by the hay monitoring system provided an average R^2^ value of 0.87 [36]. Therefore, the monitoring system-estimated yield will be, throughout this text, referred to as the actual yield. Actual alfalfa yield values of four cuts/harvests, made on 05 December, 2013 (Harvest No. 8), 16 February, 2014 (Harvest No. 9), 02 April, 2014 (Harvest No. 10) and 06 May, 2014 (Harvest No. 11) were recorded by the hay yield monitor at the time of baling (S1 Table). For each cut/harvest, yield data of 2000 geo-referenced field locations were recorded by the yield monitor for the entire study field.

### 2.6 Geostatistical analysis

**Fig 4:**
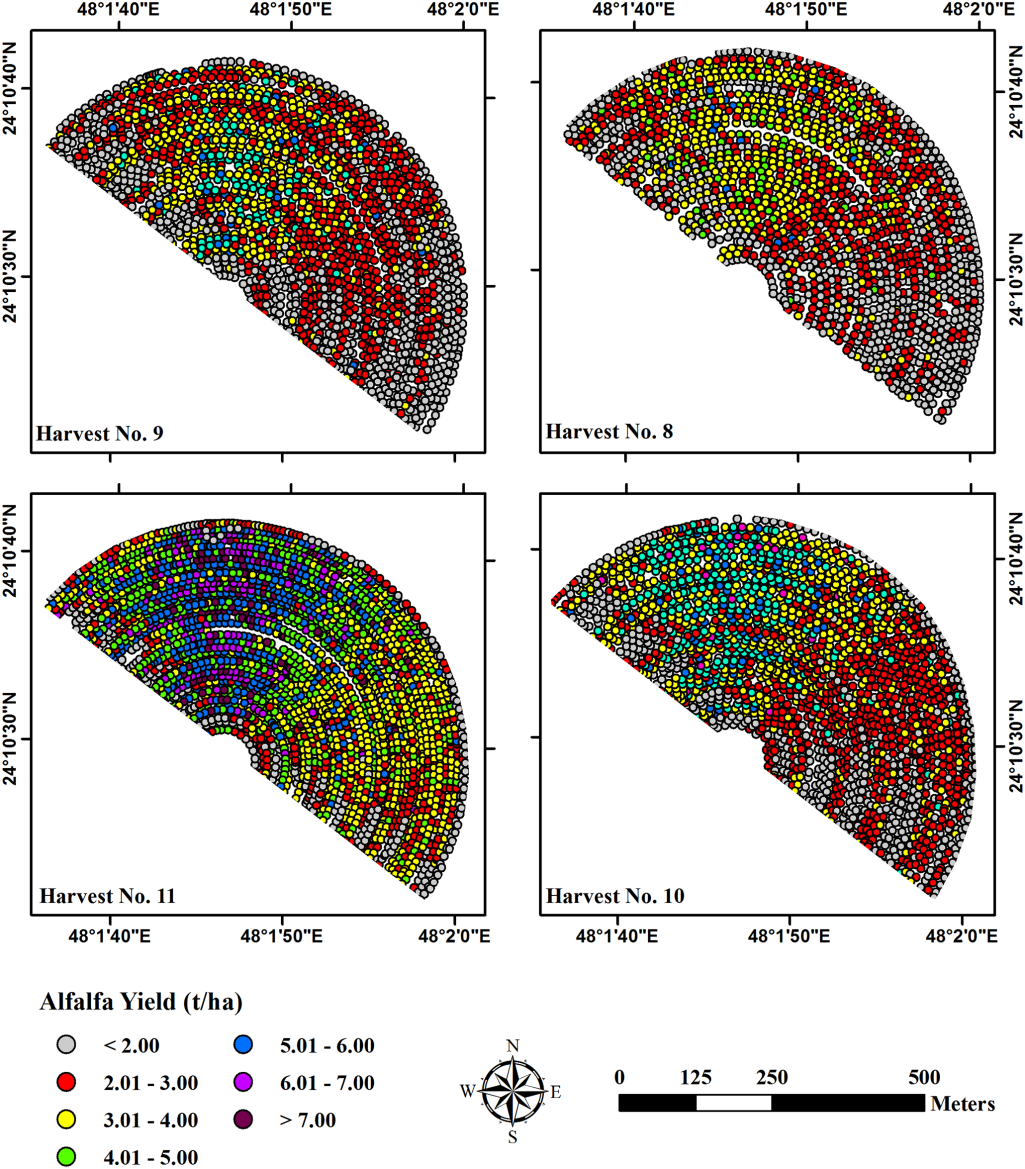
Yield monitor data points.

The spatial variability of alfalfa yield was assessed by employing geostatistical tools. Geostatistical analysis, including semivariogram model fitting and kriging procedures, were carried out using the ArcGIS software program (ver. 2010). As depicted in Fig 4, the 2000 geo-referenced yield monitor data points, representing the entire study field, were used to assess the degree of spatial variability of alfalfa yield and to create an estimated surface forming a yield map. Initially, semivariogram models were determined by using the Eq 8 [37].

$$\gamma \left(h\right)=\;\frac{1}{2N\left(h\right)}\overset{N\left(h\right)}{\underset{i=1}{\sum }}{\left[Z\left(x_{i}\right)-Z\left(x_{i}+h\right)\right]}^{2}$$ (8)

Where γ(h) is the semivariance, Z is the regionalized variable (alfalfa yield), Z(*x*_i_) is the measured sample at point *X*_i,_, Z(*x*_i_ + h) is the measured sample at point (*x*_i_ + h) and N(h) is the number of pairs separated by a distance or lag h.

The best fit to models for the collected yield data are exponential and spherical semivariogram models. Residual sum of squares (RSS), in conjunction with R^2^ values, was used to select the exact form and best fit of the semivariogram model. Subsequently, ordinary kriging interpolation with a block size of 10 m x 10 m was applied to generate the alfalfa yield map. Kriging estimation was made as per fitted semivariogram models (Eq 9)

ZX0= i=1(xi) (9)

Where Z(X_0_) is the estimate of unknown true value; λ_*i*_ is the weighted coefficient and *n* is the number of neighboring observations used in the kriging.

The accuracy of kriged maps was evaluated using cross-validation statistical methods by comparing the actual to the predicted values. Subsequently, the output of kriged image was aggregated to a grid size of 30 m x 30 m, which was similar to the spatial resolution of Landsat-8 images for the purpose of correlation analysis. The generated yield maps, derived from kriging, were analyzed against the obtained cumulative VIs and the correlation matrix was extracted using the zonal analysis tool.

## 3. Results and Discussion

### 3.1 Alfalfa yield data

For better quality of alfalfa yield, the crop was harvested at 10% bloom (harvest number 9 and 11), 30% bloom (harvest number 10) and 50% bloom (harvest number 8) stages of the alfalfa crop. Considering the climate at the time of harvesting and the behavior of alfalfa growth, the cutting schedules were adjusted among the harvests. For example, during early winter and spring seasons, the harvest was made at a crop age of 46 days. While in the winter season, the crop was harvested at an age of 73 days. However, harvesting took place at a crop age of 34 days during the spring.

The collected actual yield data sets were subjected to geostatistical analysis. Using the semivariogram, ordinary kriging was applied to the actual yield data and the properties of fitted semivariograms are presented in Table 1.

**Table 1:** Properties of fitted semivariograms.

Semivariogram Parameters	Harvest 8	Harvest 9	Harvest 10	Harvest 11
Count	1677	1977	2297	2677
Min	0.00	0.19	0.00	0.00
Max	5.86	5.97	6.68	9.96
Mean	2.32	2.45	2.86	4.01
Std. Dev.	1.01	1.07	1.24	1.69
Sample variance	1.03	1.14	1.55	2.85
Skewness (SE)	0.33(0.06)	0.33(0.06)	0.28(0.05)	0.33(0.05)
Kurtosis (SE)	0.28(0.12)	-0.10(0.11)	-0.17(0.10)	0.30(0.09)
Model	Exponential	Spherical	Spherical	Spherical
Nugget	0.02	0.54	0.67	1.43
Sill (Co+C)	0.07	1.47	2.88	4.25
Range	0.03	0.01	0.09	0.01
Residual SS (RSS)	1.24E-05	2.04E-03	0.0324	0.0287
R^2^	0.969	0.998	0.986	0.995
Proportion (C/[Co+C])	0.75	0.63	0.77	0.66

**Fig 5:**
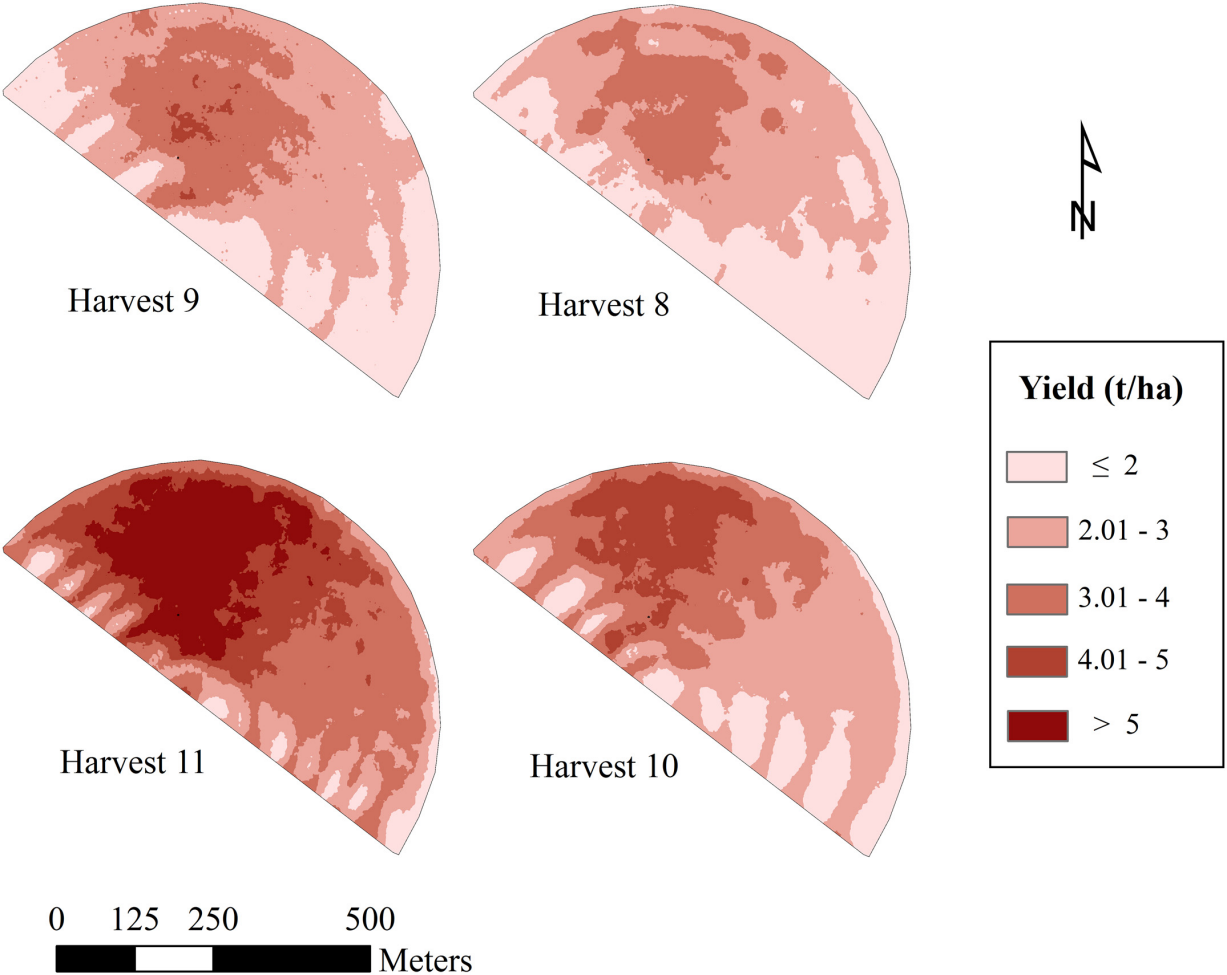
Yield monitor estimated alfalfa yield maps for the four studied harvests.

For the four cuts, actual alfalfa yield exhibited a huge variation in the productivity across the experimental field (Table 2). The spatial variability in the productivity of the crop was further illustrated by the generated alfalfa yield maps for the four harvests (Fig 5). The four yield maps showed a similar pattern of yield on spatial scale across the field. Therefore, the experimental field could be, based on productivity, categorized into three different yield zones (high, medium and low).

**Table 2:** Alfalfa actual hay yield for the four studied harvests.

**Harvest No.**	**8**	**9**	**10**	**11**
**Harvest Date**	5 December 2013	16 February 2014	2 April 2014	6 May 2014
**Yield Classes Range (t ha**^**-1**^**)**		**Area, ha (%)**		
**< 2**	8.7 (37.02)	6.7 (28.51)	4.0 (17.02)	1.0 (4.26)
**2–3**	10.7 (45.53)	10.7 (45.53)	9.8 (41.70)	3.4 (14.47)
**3–4**	4.1(17.45)	5.8(24.68)	6.2 (23.38)	8.2 (34.89)
**4–5**	-	0.3(1.28)	3.5 (14.89)	5.1 (21.70)
**> 5**	-	-	-	5.8 (24.68)
**Average yield (t ha**^**-1**^**)**	**2.32**	**2.45**	**2.86**	**4.01**

Results of the geostatistical analysis confirmed the spatial variability in the experimental field by the high significant spatial correlation between alfalfa yields for the four studied harvests. The high spatial correlation values ranged between 0.75 and 0.97 with low P-values (from 4.7E-103 to 8.9E-27) as shown in Table 3.

**Table 3:** Results of the geospatial analysis of alfalfa yield.

**Correlation (r)**				
	**Harvest 8**	**Harvest 9**	**Harvest 10**	**Harvest 11**
**Harvest 9**	0.70	-		
**Harvest 10**	0.75	0.73	-	
**Harvest 11**	0.77	0.80	0.92	-
**Total Yield**	0.86	0.87	0.94	0.97
**P value**				
	**Harvest 8**	**Harvest 9**	**Harvest 10**	**Harvest 11**
**Harvest 9**	8.9E-27	-		
**Harvest 10**	1.03E-32	2.26E-30	-	
**Harvest 11**	5.42E-35	3.07E-39	1.73E-70	-
**Total Yield**	6.08E-53	3.05E-55	2.66E-83	4.7E-103

### 3.2 Alfalfa crop vegetation indices

**Fig 6:**
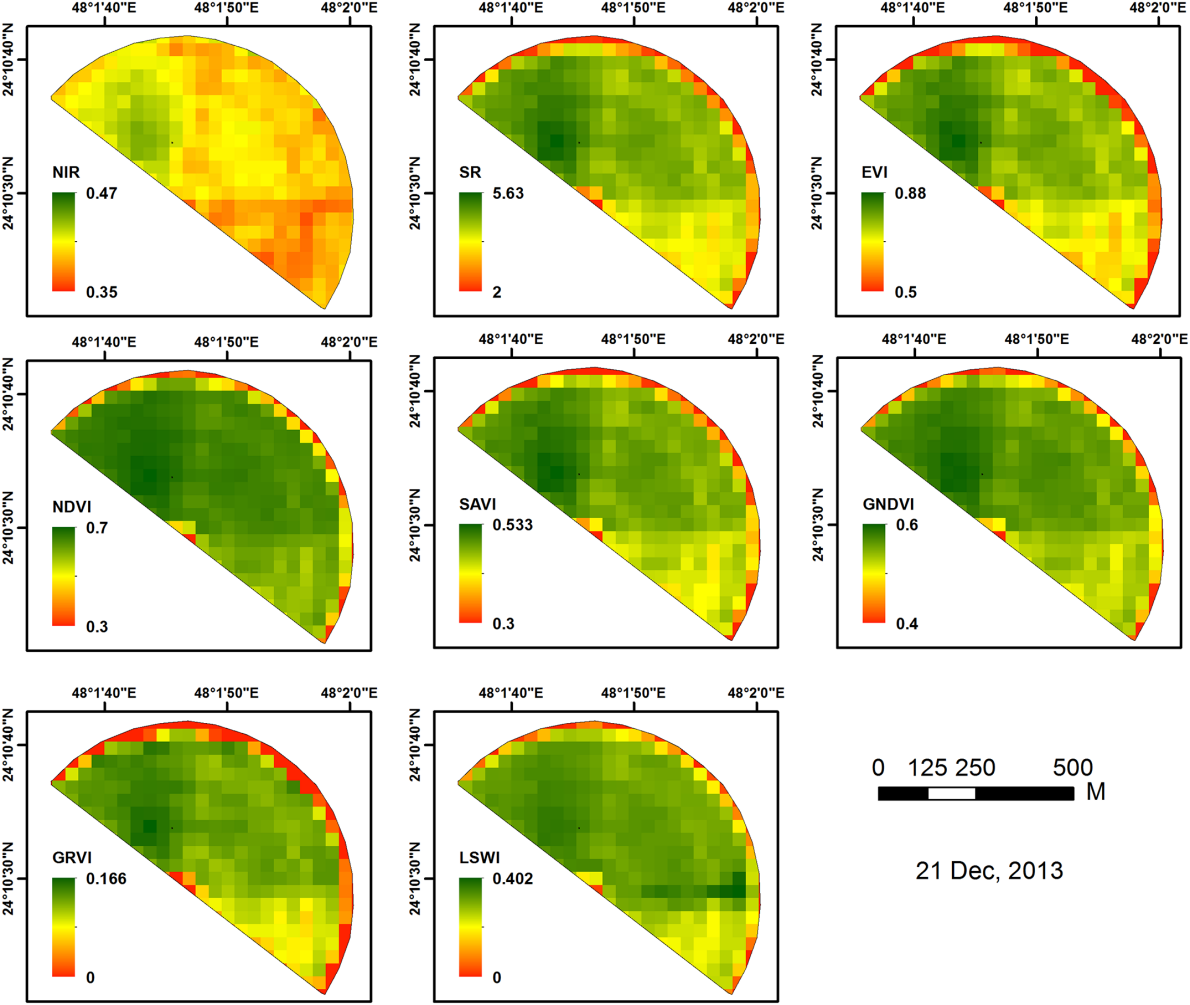
Maps of vegetation indices derived from Landsat-8 image acquired on 21 December 2013.

The acquired Landsat-8 images were analyzed for the reflectance at NIR channel; where, various vegetation indices (NDVI, SAVI, SR, EVI, GRVI, GNDVI and LSWI) were calculated at different growth stages (vegetative growth, early or late bud stage, 10% bloom, 30% bloom and 50% bloom) of alfalfa crop for the four studied harvests. The obtained maps of vegetation indices, derived from the Landsat-8 images, showed different patterns in the growth performance of the crop across the experimental field (Fig 6). On the other hand, noticeable correlations between the obtained CVIs and the corresponding actual yield were observed as the geospatial analysis was performed.(results are summarized in Table 4).

**Table 4:** Results of the geospatial analysis of CVIs against alfalfa yield.

**Correlation (r)**					
**CVIs**	**Harvest 8**	**Harvest 9**	**Harvest 10**	**Harvest 11**	**Total**
EVI	0.35	0.64	0.27	-0.04	0.39
GNDVI	0.46	0.67	0.32*	0.02*	0.47
GRVI	0.12	0.43	-0.07	-0.24	0.11
LSWI	0.33	0.57	0.23*	-0.05	0.33**
NDVI	0.35*	0.63	0.19*	-0.09	0.33**
NIR	0.49*	0.69	0.52*	0.36	0.66**
SAVI	0.43*	0.68	0.38*	0.05	0.50**
SR	0.29	0.55	0.20	-0.05	0.33
**P value**					
EVI	0.14936	0.08540	0.59248	0.17061	0.44747
GNDVI	0.15741	0.43419	0.04230	0.02848	0.06225
GRVI	0.15606	0.60578	0.05251	0.01194	0.09327
LSWI	0.87059	0.38444	0.01992	0.00091	0.00061
NDVI	0.02400	0.51578	0.00975	0.00124	0.00351
NIR	0.00735	0.08982	0.01417	0.00001	0.00002
SAVI	0.01684	0.07119	0.02984	0.00003	0.00060
SR	0.22959	0.27247	0.18250	0.00067	0.17302

* Significant at 0.05 level,

** Significant at 0.01 level.

Results of the geospatial analysis showed various trends in the spatial correlation between the CVIs and the actual alfalfa yield. Single band reflectance (NIR) and vegetation indices (SAVI, NDVI and LSWI) showed the most correlation to the actual yield as indicated by the correlation coefficients, which ranged between 0.33 and 0.66, and P-values ranging from 0.00002 to 0.00351. These four indices showed different levels of correlation to actual alfalfa yield for the four studied harvests. Among them, the NIR showed the best correlation to actual yield for, almost, all the four harvests, followed by the SAVI and the NDVI.

### 3.3 Prediction of alfalfa yield using vegetation indices

The possibility of monitoring the growth performance of alfalfa crop and predicting its yield using RS was explored. The relationship between alfalfa yield, at different crop ages, and the reflectance at the NIR channel and the selected VIs (SAVI and NDVI), derived from all acquired Landasat-8 images, was investigated (Table 5).

**Table 5:** R^2^ values describing the relationship between NIR, SAVI and NDVI and alfalfa yield.

Image No.	Date	Crop Age	Phenological Stage	NIR	SAVI	NDVI
**1**	03 November 2013	14	Vegetative	0.195	0.192	0.174
**2**	10 November 2013	21	Late bud stage	0.034	0.017	0.011
**3**	26 November 2013	37	10% bloom	0.012	0.000	0.013
**4**	05 December 2013	46	50% bloom	0.057	0.016	0.004
**Harvest 8**	**05 December 2013**	**46**	50% bloom			
**5**	21 December 2013	16	Vegetative	0.539	0.555	0.528
**6**	28 December 2013	23	Vegetative	0.466	0.391	0.159
**7**	13 January 2014	39	Dormant	0.324	0.120	0.008
**8**	22 January 2014	48	Vegetative	0.465	0.388	0.269
**9**	29 January 2014	55	Early bud stage	0.224	0.110	0.014
**10**	14 February 2014	71	10% bloom	0.376	0.458	0.561
**Harvest 9**	**16 February 2014**	**73**	10% bloom			
**11**	02 March 2014	14	Vegetative	0.137	0.040	0.004
**12**	11 March 2014	23	Early bud stage	0.248	0.154	0.018
**13**	18 March 2014	30	Late bud stage	0.146	0.061	0.070
**14**	27 March 2014	39	10% bloom	0.203	0.060	0.018
**Harvest 10**	**02 April 2014**	**45**	30% bloom			
**15**	12 April 2014	10	Vegetative	0.105	0.018	0.004
**16**	19 April 2014	17	Early bud stage	0.067	0.002	0.010
**17**	28 April 2014	26	Late bud stage	0.060	0.026	0.013
**18**	05 May 2014	34	10% bloom	0.097	0.040	0.017
**Harvest 11**	**06 May 2014**	**34**	10% bloom			

Results indicated that the studied reflectance at NIR band, SAVI and NDVI showed the highest correlation to the actual alfalfa yield at crop ages of 14 (vegetative), 16 (vegetative) and 23 (early bud stage) days for harvests number 8, 9 and 10, respectively. The highest corresponding R^2^ values of 0.54, 0.56 and 0.53 were obtained for NIR, SAVI and NDVI, respectively. At advanced crop ages, a weak correlation between NIR and actual yield was observed. This was attributed to the fact that the crop maturity or blooming stage caused an increase in the visible reflectance and altered the NIR reflectance [38, 39]. Another reason could be presented as the saturation status of the NIR reflectance as a result of a high leaf area index [25]. These results were in agreement with Trotter et al. [7], who suggested the same reason for the poor correlation, and with Ferencz et al. [13], who could not get a good correlation between alfalfa yield and VIs in small scale; however, he reported good correlations for other crops, such as corn, winter wheat, sunflower, sugar beet and winter barely. On the other hand, Borowik et al. [40] reported that seasonality shaped the relationship between NDVI and ground vegetation biomass for each habitat type. This may explain the weak relationship between alfalfa yield and VIs for harvest 11 performed in the summer compared to harvests 8, 9 and 10 conducted during either winter or spring seasons.

## 4. Conclusions

A field study was conducted to reveal the spatial variation in alfalfa yield and to explore the potential of developing an RS-based means of yield prediction using a hay yield monitor and satellite images. The specific conclusions of the study; however, can be summarized as follows:

- The hay yield monitor-generated yield maps provided a relatively definite spatial variation in alfalfa yield across the experimental field for the four studied harvests.
- Among the investigated cumulative indices, the NIR, SAVI, NDVI and LSWI showed the best correlation with alfalfa yield, as indicated by the correlation coefficients ranging from 0.33 to 0.66 and P-values from 0.00002 to 0.00351. Therefore, a non-destructive accurate means of yield prediction was provided.
- The highest correlation between studied indices and actual alfalfa yield was determined at a crop age of 14 (vegetative), 16 (vegetative) and 23 (early bud stage) days for harvest number 8, 9 and 10, respectively. On the other hand and for harvest 9, the NIR, SAVI and NDVI indices were associated with the highest corresponding R^2^ values of 0.54, 0.56 and 0.53, respectively.

## Supporting Information

S1 Table Data collected by the hay yield monitoring system. (XLSX)
